# Use of functional magnetic resonance imaging in the evaluation of neural plasticity in macular degeneration

**DOI:** 10.3389/fnins.2025.1622244

**Published:** 2025-08-28

**Authors:** Jakub Bochnička, Jan Kremláček

**Affiliations:** Department of Medical Biophysics, Faculty of Medicine in Hradec Kralove, Charles University, Hradec Kralove, Czechia

**Keywords:** age-related macular degeneration, functional magnetic resonance imaging, population receptive fields, resting-state fMRI, population connective field modeling

## Abstract

This review evaluates the use of functional Magnetic Resonance Imaging (fMRI) to investigate brain plasticity in Age-Related Macular Degeneration (AMD). An analysis of studies utilizing fMRI methods identified three primary research approaches: task-based fMRI (17 studies), resting-state fMRI (4 studies), and population receptive fields (pRF) with population connective fields modeling (pCF; 3 studies). The review outlines the principles behind each fMRI methodology and summarizes the key functional and morphological findings. Results consistently demonstrated significant structural and connectivity reorganization in the brains of individuals with AMD, suggesting that the brain undergoes adaptive responses to sensory loss. Voxel-based morphometric findings, measuring the gray matter volume loss in visual cortex, further confirm these structural changes, which appear to correlate with altered functional connectivity. These insights underscore the intricate relationship between sensory deficits and cognitive function in AMD and emphasize the potential for targeted therapeutic interventions. FMRI emerges as a vital tool in group studies for understanding the neural underpinnings of AMD and its broader cognitive implications.

## Introduction

Age-related macular degeneration (AMD) is the leading cause of visual impairment and severe vision loss worldwide. It occurs in approximately 10% of the world’s population over 60 years of age. In the population over 75 years of age, the disease occurs in up to 25% of the population (Mitchell et al., 2018). The worldwide incidence of AMD is 196 million people, of which 51 million people suffer from moderate and severe form (Wong et al., 2014). Severe visual impairment appears in the later stages of the disease in one of two forms: neovascular (wet) AMD and geographic (dry) AMD. In addition to age, other strong risk factors are darker pigmentation of the iris, cataract surgery in the past, cigarette smoking, and obesity. In clinical practice, fluorescein fundus angiography, optical coherence tomography, and autofluorescence imaging of fundus are currently widely used for diagnosis and targeting treatment. AMD causes serious difficulties in everyday life as it affects any activities requiring the distinction of fine spatial details, such as reading and recognizing faces and other objects (Stelmack et al., 2004). AMD is also associated with higher rates of cognitive impairment, worse results on cognitive tests even when not involving visually mediated tasks, and a higher risk of dementia (Zhuang et al., 2018). Retinal nerve cell apoptosis in AMD, especially in the macular region, could affect changes in brain tissue properties through transsynaptic degeneration (Prins et al., 2016). This would lead to a reduction in visual signalization from the defective macula and influence behavioral factors associated with the loss of visually dependent activities such as social interaction and reading (Zuo et al., 2020). In addition, AMD can lead to specific regional changes in the brain through various mechanisms, including loss of cognitive stimulation (a consequence of sensory impairment) and reduced feedback of regulatory signals from visual cortical areas. These changes can be subsequently followed by adaptive re-structuralizing of visual pathways and increased metabolic demand in specific cerebral regions, reflected in functional connectivity and reduced cognitive abilities (Whitson et al., 2015). In view of the above, functional magnetic resonance imaging (fMRI) is used in the research of neural plasticity in macular degeneration. It is a modern imaging method with the ability to detect dynamic local fluctuations in the ratio of oxyhemoglobin and deoxyhemoglobin. Because metabolic and neural activities are interrelated, the detected blood oxygen level dependent (BOLD) signal allows indirect monitoring of spontaneous or task-related neural activity. This review aims to overview the current studies using MRI to monitor neural plasticity in macular degeneration.

## Methodology

For this review, we searched the PubMed^1^ database using the following terms: “fMRI” + “macular degeneration.” The search was conducted on June 6, 2025, and returned 31 results. We included original studies that assessed brain plasticity in patients with macular degeneration. The inclusion criteria were: (1) studies involving human subjects diagnosed with macular degeneration, and (2) use of functional MRI (fMRI) to assess brain plasticity or reorganization. Exclusion criteria were: (1) nonhuman studies, (2) computer simulations or methodological articles without patient data, (3) studies focusing on other pathologies such as glaucoma, and (4) review articles or meta-analyses. No language restrictions were applied, and all study types were initially screened, but only original research articles were included in the final synthesis. Study selection followed a two-step process. First, titles and abstracts were screened for relevance according to the inclusion and exclusion criteria. Full texts of potentially relevant articles were then assessed for eligibility. Disagreements were resolved through discussion among authors.

Based on the results, we identified four fMRI methods used to assess brain plasticity in macular degeneration: resting-state fMRI, task-based fMRI, fMRI population receptive fields, and fMRI population connective fields. These methods are introduced shortly, and then we discuss their contribution to explanation of the disease’s impact on the brain. An overview of the studies is listed in Table 1.

**Table 1:** Summary of fMRI studies investigating the structural and functional brain changes associated with AMD covered in this review.

Study	Subjects	Method	Findings
Resting-state fMRI			
Xiao et al. (2022)	AMD: 23 (12 males, 55.7 ± 5.3 y), visual acuity < 0.5 HC:17 (9 males, 56.3 ± 5.6y)	Resting state recorded with closed eyes, analysis using rs-fMRI Data Analysis Toolkit	In AMD, higher FC in inferior frontal gyrus, superior frontal gyrus, inferior parietal lobule, rectal gyrus, and superior parietal lobule in and lower FC cerebellum posterior lobe, middle cingulate gyrus, medulla, cerebellum anterior lobe, and thalamus than in HC
Zhuang et al. (2018)	AMD: 27 (59–89 y), visual acuity 0.06–0.8 HC:27 (56–85 y)	Independent component analysis of spontaneous brain activity	Weaker functional connectivity in the primary visual cortex and lateral occipital cortex in patients
Cai and Huang (2023)	AMD: 20 (age not available) pronounced visual acuity decline and visual deformation, macular pigment disorder on the fundus photograph HC: 20 (age-matched)	Seed-based FC	Decreased FC values in the visual area, sensorimotor area, and default mode networks, different FC with ROI in the different FCS (bilateral calcarine, left supplementary motor area, left paracentral lobule)
Jiang et al. (2024)	AMD: 20 (45.15 ± 14.95 y), age >50 years! best corrected visual acuity < 0.5, presence of druse, atrophy, or neovascularisation HC: 20 (45.30 ± 13.87 y)	Voxel-mirrored homotopic connectivity (VMHC), data recorded with closed eyes	Reduced VMHC in the cuneus, superior occipital lobe, precentral gyrus, and superior parietal lobule in patients
Task-based fMRI			
Baker et al. (2005)	AMD: S1 56 y male with early-onset, visual acuity 0.06 (each eye) S2: 50 y male with an atypical form of juvenile MD, visual acuity 0.06 (left eye) HC: 4 age-matched to S1 and 4 to S2	Block design: 1—natural images with one back task, 2—words and objects foveal or peripheral presentation	Activation of the foveal cortex by peripheral visual stimuli in patients, centimeters away from the region of the cortex that responds typically to peripheral stimuli, indicating a large-scale reorganization of visual processing in human adults deprived of foveal vision
Plank et al. (2014)	AMD: 8 + 5 juvenile (47–79 y), scotoma size 10–25 HC: 12 (47–78 y)	Event-related design: texture orientation discrimination at PRL and the OppPRL	Patients with stable eccentric fixation showed better performance accompanied by a larger increase in BOLD-signal in the PRL projection zone of the early visual cortex; patients showed slower and prolonged activations with perceptual learning
Baseler et al. (2011a)	AMD: 80 y female, right damaged eye visual acuity 0.38, injections with ranibizumab at day 0, day 33, day 68, and day 165 CON: 80 y. male, bilateral disciform scarring, visual acuity 0.76	Topographic distribution and magnitude of activation in the visual cortex compared longitudinally throughout the treatment period (<1 year) and with control patients not currently undergoing treatment + clinical behavioral tests	Area of visual cortex activated increased significantly after the first treatment to include more posterior cortex that normally receives inputs from lesioned parts of the retina; subsequent treatments yielded no significant increase in activation area; the untreated control patient showed a consistent lack of significant response in the cortex representing retinal lesions
Rosengarth et al. (2013)	AMD: 9 (55–84 y) scotoma size 10–20°, duration 2–21 years HC: 7 (age-matched)	Compared activation in the respective projection zones of the PRL, the OppPRL, and the fovea in the visual cortex, namely visual areas V1, V2, and V3	Positive correlation between fixation stability and an increase in neural response in the PRL projection zones in patients during the initial phase of the training; structural changes were also found in the cerebellum of these patients following oculomotor training
Szlyk and Little (2009)	AMD: 6 (55–83 y) with bilateral geographic atrophy, visual acuity 0.06–0.26 HC: six younger (22–31 y) and six older (54–78 y)	Compared the cortical networks that underlie word recognition and processing in patients with age-related macular degeneration (AMD) with those of normally sighted control subjects	Patients with AMD demonstrate increased prefrontal and parietal activation compared with HC
Baker et al. (2008)	AMD: 5 (28–77 y) inc. juvenile with large central bilateral scotomata, visual acuity 0.06–0.26 HC: 5 (age-matched)	Block design: images presented in the fovea and the PRL with one back task	Activation of the occipital pole (corresponding to the foveal projection zone) by peripheral visual stimuli in three patients with AMD; large-scale reorganization of visual processing in AMD occurs only in the complete absence of functional foveal vision
Little et al. (2008)	AMD: 6 with bilateral geographic retinal atrophy (55–83 y), visual acuity 0.06–0.26 HC: 6 younger (22–31 y) and 6 older (54–78 y)	Block design: visually guided saccade, smooth-pursuit eye movement vs. fixation	Patients generally showed increased prefrontal cortex, intraparietal sulci, frontal and supplementary eye fields activation and decreased visual cortex activation compared with the HC; eye movement tasks required greater attention and effort in patients
Lešták et al. (2013)	AMD: 10 (1 male, 58–85 y); various degrees of bilateral impairment, no VEGF therapy HC: 9 (45–65 y)	Block design: checkerboard stimulation	Lower fMRI activity of the visual cortex was found compared with the HC group; fMRI activation did not correlate with the visual acuity
Plank et al. (2021)	AMD: 32 inc. juvenile(18 males; mean age 53.4 years, range 19–84 y); scotoma size 10–30 HC: 32 (19 males; 23–83 y)	fMRI was used for the determination of the PRL and OppPRL projection zones in V1 and oculomotor cortical areas; cortical thickness in ROIs was compared between PRL and OppPRL as well as between groups	PRL projection zone had increased cortical thickness with weak associations of fixation stability to cortical eye fields
Liu et al. (2010)	AMD: 8 inc. juvenile duration of MD more than 10 years HC: 2	Block design: PRL vs. OppPRL stimulation, full-field vs. foveal stimulation - passive, scene one back task - active	The PRL projection zone for patients had more extensive cortical representation than OppPRL; passive and active full-field stimulation did not activate the entire early visual cortex, reported as an incomplete functional reorganization in both juvenile MD and AMD
Masuda et al. (2008)	AMD: 4 juvenile (22–57 y), MD duration 10–16 years, central scotoma HC: 3 (29–35 y)	Block design: reversing checkerboard, drifting pattern, intact and scrambled faces	Responses in the LPZ while performing stimulus-related judgments reflects cortico-cortical projection in patients, not in HC; concluded as abnormal activation - not reorganization
Dilks et al. (2009)	MD: 2 (50 y, 57 y) complete bilateral loss of foveal function HC: 2 (matched)	ROI defined for all participants based on anatomical criteria at the posterior end of the calcarine sulcus with a surface area in each hemisphere of ∼200 mm2 visual stimuli presented to the same retinal location of controls as for their matched MD participant	Evident activation of the formerly foveal cortex to stimuli presented at either the PRL or an eccentric non-PRL location
Ramanoël et al. (2018)	AMD: 4 exsudative AMD (62–76 y), visual acuity 0.1–0.6. HC: 6 (age-matched)	Categorization task of indoor vs. outdoor scenes filtered in low and high spatial frequencies and modulating luminance contrast; group peak coordinates of activation in each hemisphere used to create small sphere ROIs (8 mm radius). ROIs served as the structural constraint for the analysis of the data in AMD patients and controls	The deficit for processing high spatial frequencies in AMD patients was associated with lower activity for patients in the occipital areas dedicated to central and peripheral visual fields and in the parahippocampal area, a region specialized for scene perception
Nguyen et al. (2004)	AMD: 10 (age not specified) unilateral or bilateral, HC: 5 (age-matched)	Evaluation of hemodynamic response to monocular stimulations	Significant results concerning cortical plasticity for visual perception in central vision deletion
Brown et al. (2021)	AMD: juvenile (40 y, male), bilateral absolute central scotomata (18 × 20 degrees of visual angle) HC: 10 (23–37 y, 4 males)	Retinotopic mapping, LPZ localizer analysis, high light level adaptation experiment fMRI analysis, ROI analysis	Most participants showed no evidence of stimulus-driven activation within the lesion representation, and a few individuals (22%) exhibited responses similar to a participant with juvenile MD who completed the same paradigm (without adaptation)
Ming et al. (2012)	AMD: 5 (2 juvenile, 1 male) (44–82 y), visual acuity for the better eye 0.11 ± 0.05161.0 HC: 10 (22–70 y, 6 males)	Block design in a longitudinal study: visually guided saccades and word recognition tasks in three sessions at least 4 weeks apart	HC had a higher intrasubject reproducibility of brain activation patterns than AMD (with fixed gaze, the reproducibility was higher); training visually compromised patients to use PRL improved performance and a reduction in the variability of brain activation patterns
Sunness et al. (2004)	AMD: 60 y female, bilateral geographic atrophy, visual acuity 0.4 bilaterally	fMRI retinotopic eccentricity mapping, case report	AMD caused loss of activity in the ventral visual cortex, which corresponds to the inferior retina containing the scotoma
Population receptive and connective fields			
Haak et al. (2016)	AMD: 8 juvenile (20–50 y) MD bil.central.sc. <10°, visual deprivation > 1Y HC: 12 (18–41 years)	Connective receptive fields: analysis of connection V1- V2/V3 within group compared to HC with simulated scotoma correlated pCF to fixation stability	MD < 1 Y weakened but did not destroy retinotopic connectivity between V1-V2/3
Baseler et al. (2011b)	AMD: 8 (70–90 y, 4 males), 8 juvenile (19–49 y, 3 males) bilateral lesions for at least 1 year, a stable preferred retinal locus that allows good fixation performance	Population receptive fields: wedge rotating and ring expanding stimuli (flickering checkerboard to a maximum of 15 deg)	There was no evidence of remapping of the visual cortex
Ritter et al. (2024)	AMD: 20 wet form (56–84 y), visual acuity (0.5–0.05)	Population receptive fields	Responders (11 patients) showed increased V1 activation in 3 months interval

Each row contains the study (citation and publication year), subjects (number of participants, age, and other key clinical descriptors), method (specific study paradigm), and findings (primary outcomes). The studies are subdivided into three sections: Resting-state fMRI, Task-based fMRI, and Population receptive and connective fields. (A)MD, (Age-Related) Macular Degeneration; HC, Healthy Control(s); FC, Functional Connectivity; VMHC, Voxel-Mirrored Homotopic Connectivity; PRL, Preferred Retinal Locus (the eccentric fixation point used when the fovea is damaged); OppPRL, Opposite Preferred Retinal Locus (a point contralateral or opposite to the PRL); BOLD, Blood-Oxygen-Level Dependent (the primary signal measured in fMRI); LPZ, Lesion Projection Zone (a cortical area corresponding to the damaged region in the retina); pRF, Population Receptive Field (modeling approach to assess how the visual cortex responds to different regions of the visual field); pCF, Population Connectivity Field (similar concept focusing on connectivity); V1, V2, V3, Primary, secondary, and tertiary visual cortices; VEGF, Vascular Endothelial Growth Factor; y, Years (age).

## Resting-state fMRI

If the fMRI examination is performed in a resting state and without an assigned task for the patient, it is called a resting-state-fMRI, which measures low-frequency (<0.08 Hz) BOLD signal fluctuations. In the recording, connections between individual brain regions, which are evaluated through correlations, provide information on internal functional connectivity (FC) (Raichle and Gusnard, 2005). Frontal, thalamic, and temporal cerebral regions form the so-called default mode network, which is involved in memory, emotional, and cognitive functions (Zhang and Raichle, 2010). The resting-state-fMRI is increasingly used for mapping the representations of brain functions in various diseases, such as amyotrophic lateral sclerosis (Douaud et al., 2011), traumatic brain injury (Mayer et al., 2015), stroke (Puig et al., 2018), and Alzheimer’s disease (Zhao et al., 2020). Abnormal FC has been observed in eye diseases such as glaucoma (Li et al., 2017), amblyopia (Liang et al., 2017), and strabismus (Yan et al., 2019). FC values in patients with macular degeneration are significantly increased in the superior frontal gyrus and inferior frontal gyrus, inferior parietal lobe, gyrus rectus, and superior parietal lobe, compared to the healthy control group. Decreased functional connectivity values were observed in the anterior and posterior cerebellar lobes, cingulate gyrus, thalamus, and spinal cord compared to the healthy control group. These findings of a resting-state-fMRI study by Xiao et al. in 23 AMD patients and 17 healthy control subjects may reflect compensatory changes supporting the brain activity in AMD patients with visual loss. Additionally, significant correlations between connectivity in cerebral regions and the Hospital Anxiety and Depression Scale in AMD patients were demonstrated in this study, which may suggest that anxiety and depression scores are associated with overall functional connectivity values and that abnormal neural activity may occur in areas of the brain associated with emotional activity (Xiao et al., 2022).

A study by Jiang et al. (2024) used voxel-mirrored homotopic connectivity (VMHC) via resting-state fMRI to compare brain activity between 20 AMD patients and 20 healthy controls. The findings showed significantly reduced VMHC in brain regions such as the cuneus, superior occipital lobe, precentral gyrus, and superior parietal lobule in AMD patients, which may explain their difficulties with object recognition, face recognition, and reading. Cai et al., in 20 AMD patients compared with 20 healthy control subjects, found abnormal FC within visual network, sensorimotor network and default mode network (Cai and Huang, 2023). Patients with AMD in the study by Zhuang et al. showed significantly lower functional connectivity within the V1 and lateral occipital cortex networks, but at the same time they had stronger neural-behavioral correlations with verbal fluency in the right inferior frontal gyrus and right posterior temporal regions, compared to control subjects in the corresponding age. These findings suggest that the intrinsic function of the visual cortex is impaired, possibly due to long-term visual deprivation, whereas language processing capacity in AMD patients may be well preserved in the right frontotemporal regions, in the areas with stronger connectivity. Higher functional connectivity in these regions could reflect functional reorganization (plasticity) or inherent functional advantage (cognitive reserve) in these participants (Zhuang et al., 2018).

## Task-based fMRI

Across methodological approaches, the early visual cortex plus a frontoparietal compensatory network are the most consistently interrogated regions. Tasks gravitate toward peripheral-vs-foveal visual stimulation, perceptual-learning, and eye-movement control, reflecting the two clinical hallmarks of AMD: central scotoma and reliance on eccentric fixation. Population-receptive-field and connective-field methods extend this by asking how wiring between V1 and higher tiers is re-weighted after vision loss.

When the central retina is damaged by macular degeneration, the corresponding visual representation in the primary visual cortex (V1) is partially deprived of retinal activation. Therefore, Sunness et al. (2004) using scanning laser ophthalmoscope perimetry and cortical imaging with fMRI, in a patient with geographic retinal atrophy caused by AMD, observed reduced activity in V1 in response to visual stimulation of the fovea. Ramanoel et al., in 4 patients with exudative AMD and 6 healthy controls, observed lower activity not only in the occipital areas dedicated to central and peripheral visual fields, but also in a distant cerebral region specialized for scene perception (Ramanoël et al., 2018). However, stimulation of the intact peripheral retina in the study by Masuda et al. in four patients with juvenile macular degeneration compared to three healthy control subjects, induced significant activation in V1, which is a surprising finding and could be interpreted as evidence of extensive functional reorganization of the visual cortex (Masuda et al., 2008). Similar findings were also reported by Baker et al., in two adult MD subjects with extensive bilateral central retinal lesions. In another study (2008), in 5 AMD and juvenile macular degeneration patients, they concluded the large-scale reorganization of visual processing, but only in the complete absence of functional foveal vision (Baker et al., 2008). In contrast to these findings, Baseler et al. in 16 AMD patients, with bilateral lesions for 1 year and a stable preferred retinal locus with good fixation maintained, did not observe evidence of remapping - activity in V1 in patients with macular degeneration was similar to the results of control subjects with simulated retinal lesions (Baseler et al., 2011b). The study of Brown et al. using simulated retinal lesions in healthy individuals, also did not show stimulus-driven responses in most cases (Brown et al., 2021). Szlyk et al., in six patients with bilateral geographic atrophy who were using an eccentrically located preferred retinal location, performing a three-letter and a six-letter word recognition task during fMRI, compared to the six younger and six older control subjects, reported increased brain activation in a widespread cortical network - regions identified as the frontal eye fields, superior and inferior parietal lobules, and regions within the prefrontal cortex (Szlyk and Little, 2009). Similar findings in the areas associated with attention were also reported in the study of Little et al., in 6 AMD patients and six older and six younger healthy controls (Little et al., 2008). Lestak et al., in the analysis of cortical activity in 10 neovascular AMD patients and 9 healthy controls, concluded that the visual cortex activation pattern was different and less intense if compared to healthy controls (Lešták et al., 2013). Plank et al., studying perceptual learning in 8 AMD and 5 juvenile macular degeneration patients, with scotoma sizes 10–25°, compared to 12 healthy controls, found out that patients with stable eccentric fixation showed better performance accompanied by a larger increase in BOLD-signal in the preferred retinal locus (PRL) projection zone of the early visual cortex (Plank et al., 2014). In later study (2021), in 32 AMD and juvenile macular degeneration patients, they observed possible association between the compensatory strategies used by patients with central vision loss and structural brain properties in early visual cortex and cortical eye fields (Plank et al., 2021). Rosengarth et al., in 9 AMD patients and 7 controls, found changes in the density of gray and white matter because of fixation training. They also reported a positive correlation between the increasing fixation stability and cortical response in the PRL projection zone (Rosengarth et al., 2013). Liu et al. studied cortical activation in 4 AMD, 4 juvenile macular degeneration, and two controls. The PRL had more extensive cortical representation than a retinal region with matched eccentricity, and there was no evidence for incomplete functional reorganization of early visual cortex in both juvenile macular degeneration and AMD (Liu et al., 2010). Nguyen et al. also studied cortical response in 10 patients with unilateral or bilateral AMD, and they found significant, cortical hemodynamic response to monocular stimulations (Nguyen et al., 2004). Dilks et al., in two MD patients and two healthy controls, showed clear activation of formerly foveal cortex to stimuli presented at either the PRL or an iso-eccentric non-PRL location (Dilks et al., 2009). Ming et al., in 5 MD patients and 10 controls, realized that healthy subjects showed overall higher intrasubject reproducibility of brain activation patterns than patients with macular degeneration (Ming et al., 2012).

## Population receptive fields

FMRI measures brain activity on a coarser scale (in millimeters), but it is possible to analyze fMRI signals for response features like those attributed to cells. The method of population receptive fields (pRF, quantitative model of the cumulative response of the population of cells contained within a single fMRI voxel) calculates a population receptive field model from responses to a wide range of stimuli and estimates a map of the visual field in the brain as well as other features of the neuronal population, such as receptive field size and eccentricity. The visual system implements a relationship between eccentricity and receptive field size that begins at the level of the retina. This relationship can also be observed in single-unit measurements in primates. Using fMRI and pRF modeling, it is possible to measure this relationship across multiple maps of the visual field in the cortex. In each cortical map, pRF size and eccentricity increase together. The rate of increase in pRF size varies between visual areas. Visual field maps obtained by the pRF method are more accurate than maps obtained using conventional mapping of visual field (Wandell et al., 2007). Key pRF parameters (size and position of RF) can be directly compared even when using different imaging techniques, thanks to the interpretable units specified in the stimulation frame. The important feature is the possibility of estimating receptive fields in individual subjects. Therefore, it is possible to compare pRF parameters between two subjects, in one subject under different conditions, as well as in one subject when measured by different techniques.

The pRF model implemented for fMRI has three components: a stimulus representation, a receptive field representation, and a result combining these two representations to predict the fMRI response. At each voxel, the pRF parameters are estimated to match the predicted and measured fMRI time series. The estimated size of the pRF is influenced by several undesirable factors; only some of them are explicitly modeled: eye movements, head movement, brain pulsation, and optical blur. All these factors create a bias toward a larger estimated pRF size; they add noise to an estimation of the position in the visual field. An important advantage of the method based on the pRF model is that visual field maps can be derived from responses to a wide range of stimuli (Dumoulin and Wandell, 2008).

pRF measurements in the study by Baseler et al. provided no substantial evidence of extensive remapping of cortical responses in adults with lesions in the central retina (Baseler et al., 2011b). A study by Shao et al. with the measurement of pRF in a macaque with bilateral central retinal lesion caused by juvenile macular degeneration and their results corresponded to these findings (Shao et al., 2013). However, authors Haak et al., in a study with 12 healthy participants with masking of the central part of the visual field (Haak et al., 2012), and Masuda et al., in three patients with retinitis pigmentosa and two control subjects (Masuda et al., 2010), came up with an alternative hypothesis. The sizes of the pRF near the lesion projection zone (occipital pole) are larger in patients, as well as in control subjects who were presented with stimuli that simulated the foveal lesion. Another study by Ritter et al. (2024) found that pRF mapping can effectively quantify changes in the visual cortex of patients with neovascular AMD following intravitreal ranibizumab injections. Specifically, out of 20 patients, 11 responders to anti-VEGF therapy showed increased cortical activation, while non-responders exhibited little to no change, demonstrating the potential of pRF mapping as an objective measure of therapeutic efficacy at the cortical level. Quantitative modeling using the pRF method suggests that feedback from the extrastriate cortex explains the increase in pRF size in control subjects with artificial scotomas and in patients with real lesions.

## Population connective fields modeling

Another suitable method for assessing neural plasticity is the model of population connective fields. It is a model analysis for estimating the dependence between signals in different cortical areas using fMRI. While the receptive field of the visual neuron predicts the response as a function of the stimulus location, the connective field of the neuron predicts the response as a functional expression of the activity in another part of the brain (Haak et al., 2013). Haak et al., using this model, discovered retinotopically organized patterns of functional connectivity in the projection zone of cortical lesions in eight patients with bilateral retinal lesions of at least 1 year’s duration and central scotoma due to macular degeneration. The same phenomenon was described in control subjects with simulated retinal scotoma. The results of both groups show that it is better to evaluate the integrity of visual cortical connectivity from BOLD signals that arise spontaneously than from those that are evoked by stimuli (Haak et al., 2016). Gravel et al. mapped connectivity and visuotopic fields using resting-state-fMRI in four healthy subjects in the complete absence of visual stimulation, and their results corresponded to these findings (Gravel et al., 2014). Whether changes in cerebral activity are a consequence or an adaptation to AMD remains unclear, just like the exact mechanism of these changes (Xiao et al., 2022).

## Discussion

The current review provides an overview of the use of fMRI in evaluating neural plasticity in patients with AMD. The findings from various studies highlight significant alterations in brain structure and function associated with AMD, revealing both the potential for neural plasticity and the need for further research to fully understand these changes.

The studies reviewed demonstrate that AMD leads to significant changes in both the functional and structural properties of the brain. Resting-state fMRI studies consistently show altered functional connectivity in several brain regions, including the frontal, parietal, and occipital lobes (Xiao et al., 2022). These changes suggest compensatory mechanisms that may help maintain visual processing capabilities despite retinal damage. For instance, increased activity in the frontal and parietal lobes may reflect efforts by the brain to compensate for impaired visual input through enhanced attention and cognitive control mechanisms (Zhuang et al., 2018).

Task-based fMRI studies reveal decreased activation in the primary visual cortex and other occipital areas in response to visual stimuli, which correlates with the extent of retinal damage. Interestingly, some studies report increased activation in non-visual areas, suggesting that patients may create alternative neural pathways to process visual information (Szlyk and Little, 2009; Nguyen et al., 2004; Baker et al., 2005; Liu et al., 2009). Understanding the mechanisms underlying these adaptive changes could inform the development of interventions aimed at enhancing visual function. For example, targeted cognitive or visual training programs could be designed to exploit the brain’s plasticity and improve visual outcomes in AMD patients.

Comparative evaluation of studies investigating visual cortical plasticity in AMD reveals conflicting results, much of which can be attributed to methodological variability. Baseler et al. found no evidence of large-scale remapping in early visual areas of adults with acquired macular degeneration, reporting that cortical responses in the lesion projection zone (LPZ) were not significantly different from baseline and did not extend beyond predictions based on normal retinotopic maps. Their approach relied on passive visual stimulation with expanding ring and rotating wedge stimuli, robust retinotopic mapping, and a relatively large, well-characterized sample of both AMD and juvenile macular degeneration patients and controls (Baseler et al., 2011b). In contrast, Masuda et al. observed significant task-dependent responses in the LPZ in patients with juvenile macular degeneration, but crucially, these responses were elicited only when subjects engaged in stimulus-related tasks (such as a one-back task), not during passive viewing (Masuda et al., 2008). This suggests that apparent cortical activity in the deprived zone may be driven by top-down, task-dependent feedback rather than true bottom-up remapping of visual input. Such differences highlight the impact of task design: studies relying solely on passive viewing paradigms may underestimate residual or reorganized cortical function, while those including active, attention-demanding tasks may capture additional, possibly non-retinotopic, activity. Imaging parameters, including stimulus type, field coverage, and analysis approaches, further contribute to variability in results. Additionally, participant characteristics—including age of onset, disease duration, type of macular degeneration (juvenile versus age-related), and fixation stability—can influence both the capacity for plasticity and the detectability of cortical responses. Thus, discrepancies between studies likely reflect not only true biological variability but also differences in experimental methodology, underscoring the importance of careful task design, consistent imaging parameters, and comprehensive characterization of participants in future research.

Longitudinal studies examining the progression of both AMD and cognitive decline could provide valuable insights into whether AMD is a marker of more widespread neurodegeneration. Using voxel-based morphometry, in 9 patients with AMD, with a homonymous scotoma lasting at least 3 years, with a diameter of at least 10° in at least one quadrant, Boucard et al. found a reduction of gray matter mainly near the occipital pole (primarily in the left hemisphere), especially around the posterior part of the calcarine sulcus (Boucard et al., 2009). Hernowo et al. confirmed these findings in a group of 24 patients with AMD and 34 with juvenile macular degeneration with a scotoma diameter of 14° (range 4–25) and additionally described a reduction of white matter in the geniculocalcarine tract, in the visual cortex and surprisingly also in the frontal lobe (Hernowo et al., 2014). Shen et al., in 18 neovascular-AMD patients, also described significantly lower gray matter volume in the right inferior frontal gyrus, temporal pole of left superior temporal gyrus, left superior temporal gyrus, left middle frontal gyrus, left anterior cingulate and para cingulate gyrus (Shen et al., 2021). Degeneration in the post-geniculate part of the visual pathways of patients with AMD can be explained by a reduction in input stimuli from the visual field toward the visual cortex. However, the described volume reduction of white matter in the frontal lobe of patients with AMD creates a neural correlation of an earlier hypothesis about the association between AMD and mild cognitive impairment or Alzheimer’s disease (Klaver et al., 1999). Woo et al., in a cohort of 170 patients with AMD, using the evaluation of cognitive functions through 15 psychological tests, revealed that AMD and Alzheimer’s disease share several clinical and pathological features (Woo et al., 2012). This supports the opinion of Kaarniranta et al. that AMD could be a manifestation of a more general neurodegenerative disease, where the primary symptom of the disease may be the degeneration of the visual pathway (Kaarniranta et al., 2011). However, the findings of the different genetic backgrounds of both diseases were published by Proitsi et al. after genotypic analysis of four large cohorts of patients from genetic center databases (Proitsi et al., 2012). In addition, Keenan et al. comparing a cohort of 65,984 AMD patients with a reference cohort of more than 7.7 million people, demonstrated that the chance of developing Alzheimer’s disease after AMD was not statistically significantly different from random chance (Keenan et al., 2014).

The distinction between ocular and neurodegenerative disease is essential for determining the correct therapy. Baseler et al., in a case-report study, found out that the area of the visual cortex activated increased significantly after the first treatment of neovascular AMD to include more posterior cortex that normally receives inputs from lesioned parts of the retina. Subsequent treatments yielded no significant further increase in activation area (Baseler et al., 2011a). Eckardt and Eckardt conducted studies on macular translocation (Eckardt and Eckardt, 2002), and van Zeeburg et al. studied retinal pigment epithelium transplantation (Van Zeeburg et al., 2012), aimed at restoring visual function in patients with AMD. These studies have shown different and not always successful results in improving visual functions. Distant and near vision improved with macular translocation, but metamorphopsia appeared in one patient and geographic retinal atrophy in another. Epithelial transplantation increased visual acuity, but only in about a fifth of patients. However, if AMD turns out to be a neurodegenerative disease, then the neurodegenerative component may be responsible for the sometimes-weak effects of the treatment.

A comprehensive assessment of fMRI findings in AMD should be accompanied by consideration of their structural and neural correlates, which are now accessible noninvasively. Advances such as optical coherence tomography (OCT) enable detailed structural imaging of the retina, while the brain’s rapid temporal dynamics can be captured through electroencephalography (EEG) or visually evoked potentials (VEP). Despite the potential of these complementary modalities, truly integrative and longitudinal study designs remain rare.

Technically, co-registration with OCT is straightforward, as both fMRI retinotopic maps and OCT fundus images can be aligned using a shared polar coordinate system (eccentricity × polar angle). EEG provides millisecond-level temporal precision, allowing researchers to probe whether neural compensation occurs via accelerated peripheral input processing or through the recruitment of higher-order feedback mechanisms. However, precise co-registration across time domains is more challenging; yet, over the past three decades, methods have matured to enable meaningful integration of EEG and fMRI data (Warbrick, 2022).

This multimodal approach is particularly valuable in tracking longitudinal changes in cortical remapping, especially when evaluating interventions aimed at enhancing visual plasticity, such as pharmacological treatments or noninvasive brain stimulation. For instance, in a six-month series of ranibizumab treatments, responders exhibited a 29% expansion of the active V1 area—a change that paralleled OCT-detected fluid resolution and predicted visual outcomes more reliably than standard acuity tests. These findings position multimodal fMRI as a sensitive biomarker for disease progression and therapeutic response (Ritter et al., 2020). Furthermore, independent reproducibility studies have demonstrated sub-millimeter test–retest precision for retinotopic activation maps, validating their application in detecting within-subject changes over months or years. This enables detailed modeling of plasticity kinetics in both neurodegeneration and rehabilitation contexts (Ming et al., 2012).

## Limitations

There are notable methodological variations that may account for differences in findings. For instance, the type of fMRI (resting-state vs. task-based), the nature of the visual tasks, the characteristics of the patient populations (e.g., age, severity of AMD), and the relatively small sample sizes in many studies can influence results. These limitations highlight the need for larger, multicenter studies and standardization in the design to facilitate comparison and synthesis of findings to validate and explore the neural plasticity in AMD.

## Conclusion

Functional magnetic resonance imaging is an important method in scientific research of central nervous system function. Its use in patients with macular degeneration shows ongoing functional reorganization in the visual area and changes in connectivity, which indicate the reorganization of frontal-temporal networks. This is an important basis for further research into effective therapeutic options and procedures in the treatment of this widespread disease. Morphological changes appear mainly in the primary visual area and visual pathways, where there is a volume reduction of brain tissue. Increased connectivity is described in the frontal and parietal lobes when using the mentioned fMRI methods - but the hypothesis, whether these changes are a consequence of the disease, adaptation of the brain to defective stimuli, or a correlate of a neurodegenerative disease, must be verified by further research.

## Footnotes

^1^ https://pubmed.ncbi.nlm.nih.gov
